# A Pilot Mixed-Methods Study to Establish the Clinical Usefulness of a Chronic Pain Profile (CPP) for Pain Management

**DOI:** 10.3390/jcm12165374

**Published:** 2023-08-18

**Authors:** David R. Axon, Darlena Le, Jonathan Chien

**Affiliations:** 1 Department of Pharmacy Practice & Science, R. Ken Coit College of Pharmacy, University of Arizona, 1295 N Martin Ave, P.O. Box 210202, Tucson, AZ 85721, USA; darlenal@arizona.edu (D.L.); jonathanchien1@arizona.edu (J.C.); 2 Center for Health Outcomes and PharmacoEconomic Research (HOPE Center), R. Ken Coit College of Pharmacy, University of Arizona, 1295 N Martin Ave, P.O. Box 210202, Tucson, AZ 85721, USA

**Keywords:** pain, pain management, qualitative research, clinical research

## Abstract

The Chronic Pain Profile (CPP) was developed as a tool for patients to document types and levels of use for all pain management strategies used. This pilot mixed-methods (quantitative and qualitative methods) study aimed to assess the perceived clinical usefulness of the CPP and identify potential areas of difficulty using the CPP among a sample of pharmacists. Data were obtained from an online survey of pharmacists licensed to practice in Arizona. Quantitative analysis included assessing the clinical usefulness of the CPP using 10 numerical items (scores ≥50% = useful), 5 ordinal items (scores ≥ 4 out of 5 = useful), and 11 open-response items. Qualitative analysis was conducted by two independent researchers who coded the comments and identified key themes through consensus. Data were collected for 33 individuals. Mean usefulness scores ranged from 66.6 ± 22.4 to 80.9 ± 23.5, and three of the five ordinal items had a median score ≥ 4. Three key themes (and subthemes) were identified: favorable features of the CPP, which included promoting patient advocacy and saving time when accessing pain information; using the CPP, which included evaluating of the effectiveness and appropriateness of the pain management approach and identifying gaps in patient knowledge; and limitations of the CPP, which included absence of customization, interpretation issues, complexity and wording issues, and concerns of internal consistency and reliability. This pilot study provides initial evidence of the CPP’s clinical usefulness that could ultimately be used to help manage pain and improve health outcomes. Further analyses are needed to assess the CPP’s validity and explore its use in wider populations of patients with pain.

## 1. Introduction

Chronic pain is defined as “ongoing or recurrent pain, lasting beyond the usual course of acute illness or injury or more than three to six months, and which adversely affects the individual’s well-being” [1]. Chronic pain is a prevalent condition; estimates of its prevalence vary in the literature (due to different definitions and study designs) from 25 million United States (US) adults in 2012 [2], 50 million US adults in both 2016 [3] and 2019 [4], and 51 million US adults in 2021 [5], and it contributes to frequent healthcare use, high healthcare costs, and poorer health outcomes [6,7,8]. 

Many pain management strategies exist, including prescription and nonprescription medications and opioid and nonopioid medications [9], as well as medical [10], physical [11], psychological [12], and self-initiated strategies [13]. The use of multimodal (concurrent use of medications with different modes of action) and multidomain (use of strategies from different domains of therapy, e.g., medications and massage) strategies has been recommended [14], as has the need for studies investigating nonpharmacological pain management strategies [15]. Adequate pain management requires a holistic approach that includes knowing the exposure to all strategies and using them at therapeutic levels [2]. 

One study reported that individuals with chronic pain used a mean of 12.6 ± 4.6 pain management strategies [16]. Given the considerable number of strategies used to manage pain and variation in exposure (dose) to these strategies, the exposure multimodal (Xm2) score was developed to standardize all exposures using a 5-point scale based on the maximum recommended dose for each strategy [16]. Briefly, a score of 1 represents use of <25% of the maximum recommended dose, and a score of 5 represents 100% [16]. Scores > 5 indicate use beyond the maximum recommended dose. To present personalized Xm2 scores and related data about an individual’s pain management strategies in a meaningful way for both patients and healthcare professionals, the Chronic Pain Profile (CPP) was developed. 

This study aimed to assess the perceived clinical usefulness of the CPP and identify potential areas of difficulty using the CPP using a survey design. The findings of this study should help inform any necessary revisions to the CPP and contribute to the development of a clinically useful tool to help individuals manage their pain more effectively.

## 2. Methods

The study was conducted in accordance with the Declaration of Helsinki and approved by the Institutional Review Board (or Ethics Committee) of The University of Arizona (protocol code 1911159728, 5 December 2019). Informed consent was obtained from all subjects involved in the study.

**Figure 1:**
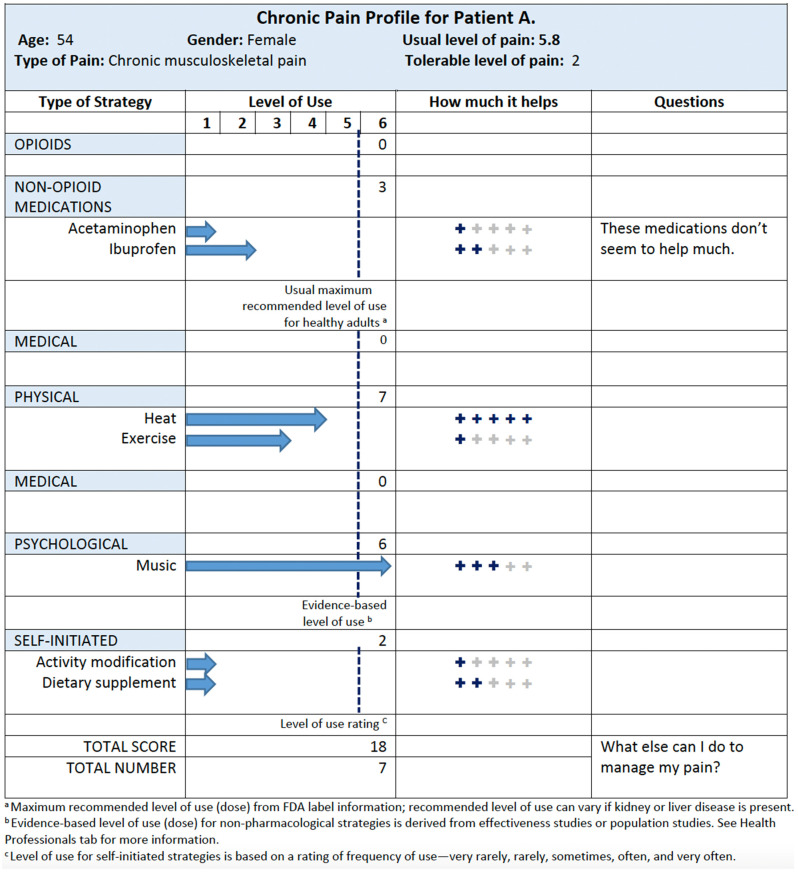
Example Chronic Pain Profile (CPP). This figure provides an example of the Chronic Pain Profile (CPP) for a fictional patient. Demographic and pain characteristics are provided at the top of the CPP. Each of the four sections in the CPP provides information on the types of strategies used, their level of use, how much the strategy helps, and a space for users to note down any questions they have for their provider. Footnotes are provided at the bottom of the CPP to further explain the content.

An example CPP is provided in Figure 1. Characteristics of the patient (e.g., name, age, and gender) and characteristics of their pain (e.g., type of pain, usual level of pain, and tolerable level of pain) are presented at the top of the profile. The main body of the profile records the strategies used to manage pain. These strategies can include medications (e.g., opioids, prescribed medications, and over-the-counter medications), medical strategies (e.g., visiting a specialist and surgery), physical strategies (e.g., exercise and heat), psychological strategies (e.g., counselling and prayer), and self-initiated strategies (e.g., dietary modification). The level of use (calculated and reported as an Xm2 score as described above) and how much the strategy helps (as indicated by zero to five stars) are presented alongside each strategy. There is also an area where the individual can note any comments or questions they have about a particular strategy. The CPP was designed to be used by individuals with pain to help them manage their own pain and in consultation with healthcare professionals (e.g., physician, pharmacist, and physical therapist) to help optimize health outcomes.

This study included pharmacists because their knowledge should enable them to respond to detailed questions about use and dose/exposure of pain management strategies, their education teaches them about the need to rigorously test new healthcare technologies (in this case an instrument rather than a medication), and they are potential users of the CPP, either as healthcare professionals working with pain patients or as a person with pain themselves. Licensed Arizona pharmacists whose email addresses were registered with the State Board of Pharmacy in 2020 were eligible for this study.

The questionnaire first contained six items that asked about the usefulness of the CPP content (0–100 scale) followed by two open-response items. The questionnaire then asked how easy it was to understand the information on the CPP (0–100 scale), which was followed by five items that asked how easy it was to understand each of the five CPP sections (5-point Likert scale) and five open-response items to comment on each section. Next, participants were given a link to the CPP web page and asked to rate the overall appearance of the webpage, how easy the webpage was to use, and how easy it was to find information on the webpage (on a 0–100 scale). This was followed by an open-response item to comment on the appearance, function, or content of the webpage. Finally, 11 items collected data on demographic characteristics and professional experience. See Appendix A. 

The questionnaire was administered using Research Electronic Data Capture (REDCap; Vanderbilt University Medical Center, Version 8.10.0, Nashville, TN, USA) over six weeks between January and March 2020. An initial email was sent to eligible participants in January 2020 that described the study and invited them to participate. One week later, eligible participants received an email with a link to the survey. Reminder emails were issued two and four weeks later. At the start of the questionnaire, participants read a consent form and indicated their willingness to participate in the study. Participants who completed the questionnaire could download a certificate of appreciation.

Data analysis involved both quantitative and qualitative methodologies (i.e., mixed methods) to gain a more holistic assessment of the CPP’s clinical usefulness. Demographic characteristics and professional experience variables were summarized using frequencies (percentages) for nominal data and means (standard deviations, SD) for normally distributed continuous data. Usefulness items with normally distributed continuous data were summarized as mean (SD). Item mean scores ≥ 50% indicated evidence of the CPP’s usefulness. Usefulness items with ordinal data were scored as follows: not at all easy = 1, somewhat easy = 2, easy = 3, quite easy = 4, and very easy = 5, and they were summarized as median (interquartile range, IQR). Item median scores ≥ 4 indicated evidence of the CPP’s usefulness. For qualitative analysis, two trained independent reviewers (DL and JC) iteratively coded the responses obtained from the open-response items. The two reviewers met to compare code lists, and a third reviewer (DRA) facilitated the resolution of discrepancies through discussion. Inter-rater reliability was calculated. Common themes were identified from the code lists, and representative quotes were selected to exemplify each theme. 

## 3. Results

Data were collected for a total of 33 pharmacists licensed to practice in the state of Arizona in 2020. The majority of participants were female (64%), ≥50 years of age (54%), white (64%), married (57%), resided outside of Arizona (52%), were employed (85%), had completed continuing education on pain management (89%), and did not currently or previously work at a practice site that helps patients manage pain (73%). Participants were approximately evenly split between primarily working at community (30%), hospital (33%), and other (37%) practice sites. Participants had practiced pharmacy for a mean of 20.6 ± 13.7 years and reported a mean score of 38.4 ± 26.4 (on a 0–100 scale) when asked how frequently their job responsibilities involved helping patients manage pain. 

Table 1 displays participant’s responses to the quantitative items. On average, all six items about the perceived usefulness of the CPP content were scored above the midpoint, with mean values ranging from a low of 67.9 ± 24.1 to a high of 80.9 ± 23.5. The mean score for how easy it was to understand the information in the CPP was also above the midpoint, at 69.2 ± 23.5. Among the five items that assessed how easy it was to understand each section of the CPP, the median score was 4 for three of the items and 3 for two of the items. On average, all three items about perceptions of the CPP webpage were scored above the midpoint, with mean values ranging from a low of 66.6 ± 22.4 to a high of 71.0 ± 24.9.

Table 2 displays the themes identified from thematic (qualitative) analysis of the open-response items, along with representative quotes. The two independent code lists had a 93% level of agreement, with differences resolved through discussion until consensus was reached. Three primary themes were identified and are described below: (1) favorable features of the CPP; (2) using the CPP; and (3) limitations of the CPP.

Theme 1: Favorable Features of the CPP

Two groups of favorable features of the CPP were identified: (1) promotes patient advocacy and (2) saves time when accessing pain information.

Theme 1.1. Promotes Patient Advocacy

Participants commented that the CPP could be a useful resource for patients to facilitate their active participation in their pain management approach, help improve their understanding of their pain management, track their current approach, and self-assess and identify improvements to their pain management approach. In particular, several participants mentioned how the ‘Questions’ section could serve as a reminder for patients to ask questions and allows them more time to formulate those questions.

Theme 1.2. Saving Time when Accessing Pain Information

Participants indicated the CPP could serve as a way to access all relevant information about a patient’s pain management approach in one document, which could save time for the provider during the appointment. The profile would also help guide them on what topics to focus on during conversations with patients. One participant contrasted the CPP to SOAP notes and described the profile as saving time when healthcare providers initially screen the patient’s pain regimen while also making sure all patient needs are met.

Theme 2: Using the CPP

Participants comments about how the CPP could be used in clinical practice were organized around two subthemes: (1) evaluation of the effectiveness and appropriateness of the pain management approach and (2) identification of gaps in patient knowledge.

Theme 2.1. Evaluation of the Effectiveness and Appropriateness of the Pain Management Approach

When examining the example CPPs, participants described how they could use the CPP as a pain medication reconciliation tool, for instance, to identify issues with medication doses and suggest a resolution. Participants used the CPP to gain insight into how effective the pain management was for the patient. Participants also used the CPP to identify and query the appropriateness of some medications used by patients. Furthermore, participants used the CPP to review the patients’ pain management approach and identify areas where more information is needed to complete their assessment and make sure the most appropriate strategies are in place. Participants were then able use information in the CPP to suggest alternative/additional pain management strategies, including both pharmacological and nonpharmacological strategies.

Theme 2.2. Identification of Gaps in Patient Knowledge

Participants used the CPP to help identify knowledge gaps and educational opportunities for patients to optimize their pain management approach. Participants commented that such knowledge gaps and educational opportunities offered an opportunity to enhance patient–provider communication, medication counselling, and ultimately, therapeutic decision making for chronic pain management.

Theme 3: Limitations of the CPP

Participants perceived four areas of difficulty when using the CPP that could be improved: (1) absence of customization features limiting comprehensiveness; (2) interpretation; (3) complexity and wording; and (4) concerns of internal consistency and reliability.

Theme 3.1. Absence of Customization Features Limiting Comprehensiveness

Some participants commented that the inability to add custom features, sections, and subsections could hinder the completeness of the data collected and CPP use. Many participants reported that the CPP was currently insufficient for capturing a complete medical history for pain management and requested the option of adding additional information. Participants reported that including customization features such as demographic options, pain treatment categories, and information displayed would help collect additional patient information that could provide a more complete medical history of the patient’s pain and assist with developing patient-centered pain recommendations.

Theme 3.2. Interpretation

Participants indicated that the extent of CPP use may depend on healthcare providers’ familiarity and experience with the CPP.

Theme 3.3. Complexity and Wording

Some pharmacists reported that the tool was too complex and confusing because of the length of descriptions. Other participants reported that overlapping pain management categories created confusion and impaired usability.

Theme 3.4. Concerns of Internal Consistency and Reliability:

Some pharmacists questioned the CPP’s usefulness because of the measurement scale(s) used. Some pharmacists disagreed with the current pain scale ratings because of the subjectivity and difficulty of interpreting pain severity. Finally, one participant expressed concern that the availability of the CPP would increase the risk of patient abuse and falsifying information.

## 4. Discussion

This pilot study provides initial evidence of the CPP’s perceived clinical usefulness from a sample of pharmacists licensed in the US State of Arizona. Quantitative analyses demonstrated all ten interval/ratio items were scored above the midpoint, and three of the five ordinal items scored ≥4. We stipulated a priori that mean scores above the midpoint (interval/ratio items) and median scores ≥ 4 (ordinal items) would provide evidence of clinical usefulness. Therefore, these findings provide good evidence for the CPP’s clinical usefulness.

In qualitative analyses, the sample of pharmacists perceived the CPP would enable individuals to be actively involved by monitoring and recording their pain management progress. This is useful because patients are often responsible for self-managing their pain due to poor healthcare access or requiring several specialists [2]. This is also useful because we already know pain management is burdensome and complex given that a mean of 12.6 ± 4.6 pain management strategies were used in a recent study [16]. These findings suggest the CPP could facilitate pain self-management by enabling patients to review their pain management strategies, identify or try new approaches, and help patients feel more in control of their pain management regimen. 

This sample of pharmacists perceived the CPP could improve healthcare providers’ accessibility to patient-specific pain management information. This is useful because obtaining relevant information about an individual’s pain management is often the first step to understanding and improving pain management, which can be difficult to comprehensively accomplish given the large number and types of pain management strategies used [5,8]. 

Patients often have negative experiences with primary care providers due to limited time or feeling intimidated to ask all their questions [17]. The “questions” section of the CPP may therefore help pain patients organize their thoughts and prepare questions in advance to alleviate the pressure of initiating questions themselves and optimize the (often limited) time available with their provider. Having time to discuss treatments with providers, which affirms the importance of patients being actively involved in their pain management, is associated with greater patient satisfaction [18]. 

This sample of pharmacists identified two major uses of the CPP: evaluating the effectiveness and appropriateness of pain management approaches and identifying gaps in patient knowledge. The CPP may serve as a reconciliation tool to take a complete pain-related medical history, particularly among complex patients with multimorbidity or using multiple strategies [13,16]. This may be useful given that a previous study found a complete basic medical history was not obtained for 25% of patients [19]. Pharmacists were able to use the example CPP to assess the appropriateness of medication dosages and indications. This is useful because pharmacist-led medication reconciliation has been found to reduce adverse drug-event-related hospital visits, emergency department visits, and hospital readmissions [20]. This sample of pharmacists were also able to use the example CPP to identify additional/alternative pain management strategies, which included recommending nonpharmacological strategies. This is useful because it aligns with best-practice recommendations for an integrated multidisciplinary approach to pain management interventions [21]. These scenarios enabled pharmacists to identify gaps in patients’ knowledge and offered opportunities for patient education. 

The CPP could be incorporated into the medical record to enable other healthcare professionals to access it and facilitate interprofessional communication [22]. This is useful as individuals with pain are likely to receive treatment from several healthcare professionals (e.g., primary care provider, pain specialists, pharmacists, and others) who could benefit from accessing the patients’ latest pain management regimen. 

Finally, qualitative analyses identified perceived limitations of the CPP, which included lack of customization, interpretation, complexity and wording, and concerns about internal consistency. This is useful as it provides an opportunity for refinement before recommending future widespread use. We suggest a balance is needed with potential refinements, e.g., between customization and standardization or being too complex/wordy versus ease of interpretation. Typically, as the instrument becomes more personalized, the level of standardization decreases. Greater personalization and less standardization may lead to greater complexity and difficulty with interpretation. To address this, it is possible that additional data or sections could be included (and left blank by patients if the section is not applicable to them) to enable additional data collection, while training could be provided to explain its use and interpretation. 

Although previous research has established evidence of validity for the Xm2 algorithm [16], additional research is warranted to assess the CPP’s internal and external validity. Future research is also warranted to assess the reliability and internal consistency of the CPP. Furthermore, the clinical usefulness, validity, and reliability of the CPP should be assessed in larger samples of healthcare professionals, such as physicians, nurse practitioners, physical therapists, etc. (i.e., those who are likely to use the CPP as a provider), and among the population of patients with pain (i.e., those who are likely to use the CPP as patients).

Study limitations included features of survey design (e.g., assuming participants understood the questions and responded honestly) and the small convenience sample of pharmacists from one US state (which restricts generalizability). Given that the survey was sent to all licensed Arizona pharmacists whose email addresses were registered with the State Board of Pharmacy in 2020, the response number of 33 represents a very low response rate, which may have biased the findings. It is possible that only the pharmacists with an interest in this topic responded, which may also have biased the findings. Finally, responses from pharmacists may be different from other healthcare professionals, e.g., physicians who may have been equally well qualified to comment on the CPP, which may be another source of bias.

## 5. Conclusions

In conclusion, this study provides initial quantitative and qualitative evidence of the CPP’s clinical usefulness from a small sample of pharmacists. These findings offer insight into how these pharmacists perceived the CPP could ultimately be used by individuals with pain to better manage their condition and improve health outcomes. Further refinement and analyses are needed to assess the CPP’s validity and to explore its use in wider populations of patients with pain. 

## Figures and Tables

**Table 1:** Participant’s perceptions of the Chronic Pain Profile (CPP).

Usefulness of CPP Content (Scored 0–100)	Mean ± SD
The strategies used to manage pain are divided into 5 categories: medications, medical, physical, psychological, and self-initiated strategies. How informative are these categories in the context of pain management? (N = 30)	72.6 ± 21.7
A level of use score is provided for each strategy listed based on the amount used and the recommended level of use. How helpful is the level of use score when thinking about modifying pain management strategies? (N = 29)	67.9 ± 24.1
How useful is it to have a patient’s rating of how much a specific strategy helps them manage their pain? (N = 29)	80.6 ± 23.1
How useful is it to have patient questions related to each strategy included in the Chronic Pain Profile? (N = 27)	80.9 ± 23.5
If a patient brought a pain management profile similar to the example to their appointment with you, how useful would you rate the profile? (N = 30)	78.3 ± 25.2
How much would having a patient complete a Chronic Pain Profile before their appointment save time during the appointment? (N = 28)	70.0 ± 27.3
CPP (scored 0–100)	Mean ± SD
How easy was it to understand the information in the Chronic Pain Profile? (N = 29)	69.2 ± 23.5
How easy each section of the CPP is to understand (scored 1–5)	Median (IQR)
Patient information (N = 32)	4 (2)
Types of strategy (N = 32)	4 (2)
Level of use (N = 32)	3 (2)
How much the strategy helps (N = 32)	3 (2)
Questions (N = 31)	4 (2)
CPP web page (scored 0–100)	Mean ± SD
How attractive would you rate the overall appearance of the web page? (N = 27)	69.7 ± 18.0
How easy was the web page to use? (N = 28)	71.0 ± 24.9
How easy was it to find the information that you wanted? (N = 28)	66.6 ± 22.4

N indicates the number of respondents for each item (some items have missing data). Ordinal items scored as follows: not at all easy = 1, somewhat easy = 2, easy = 3, quite easy = 4, and very easy = 5, and they are summarized using median (IQR). IQR = interquartile range. SD = standard deviation.

**Table 2:** Themes and subthemes identified from qualitative analysis of open-response items.

Theme/Subtheme	Representative Quote
1. Favorable features of the CPP	
1.1. Promotes patient advocacy	“It is a good tool to keep track of what has been tried and what level of results were noticed.” (P23)
	“In order to get them thinking about their pain and evaluate past strategies to create a new strategy if the current regimen is not providing sufficient pain management.” (P25)
	“I know they may have questions or concerns that are not being addressed and they may be able to articulate their feelings more clearly under the question section.” (P17)
1.2. Saving time when accessing pain information	“It would be nice to see what the patient is using or has already used to go further with the next recommendations for pain control. It would save consultation time to see this document.” (P29)
	“It is useful for healthcare professionals to understand patient’s pain management without digging through the SOAP notes. Also, this profile format would help healthcare professionals to ask all the necessary questions to assess how patients are doing with their pain management … due to a limited time for patient appointments, healthcare professionals sometimes forget to ask questions.” (P1)
2. Using the CPP	
2.1. Evaluation of the effectiveness and appropriateness of the pain management approach	“Patient is not using an effective dose for Tylenol and Ibuprofen. Would recommend Tylenol 500 mg at 1–2 every 4–6 h and Ibuprofen 200 mg 2–3 every 6 h.” (P31)
	“It shows me all the different strategies she is using to help with her pain and how well they are working.” (P3)
	“Why is patient taking two different muscle relaxants?” (P17)
	“Would be helpful to know what neuropathic pain due to such as diabetes, amputation, Raynaud’s, etc. Each of these might be treated slightly different. Hydrocodone is not the most effective for neuropathic pain.” (P32)
	“Might try either amitriptyline or duloxetine since this patient may also be somewhat depressed.” (P32)
	“Her pain responds well to heat. Maybe there is an exercise she can do in a warm environment, e.g., sauna, hot yoga, swimming, etc.” (P12)
2.2. Identification of gaps in patient knowledge	“Metoprolol is not for pain; patient education is needed. Patient is using narcotics that are not very effective for neuropathic pain.” (P8)
	“There may be other counseling options (specific counseling/therapy for patients in pain) that I could direct him to; there’s also an opportunity to maximize his medications and talk about possible changes in regimen.” (P13)
3. Limitations of the CPP	
3.1. Absence of customization features limiting comprehensiveness	“Having more information on the history of pain should help healthcare professionals’ terms of understanding patients’ pain.” (P1)
	“Race/ethnicity info would be helpful to gauge cultural aspects of pain and treatment of pain.” (P27)
3.2. Interpretation	“Interpretation of the profile would need some practice.” (P11)
3.3. Complexity and wording	“Need more balance between words and visual. I like the visuals and can read through the text associated with the profiles. I don’t need it to be explained to me.” (P12)
	“Self-initiated’ could be a bit confusing. If I start taking Tylenol on my own without medical advice, is that a self-initiated strategy? If I sit in a hot tub, is that self-initiated or physical?” (P23)
3.4. Concerns of internal consistency and reliability	“May be nice to include a pain scale so that this can be standard from patient to patient.” (P8)
	“Would breed ways to beat the system for abuse.” (P28)

## Data Availability

Data are available from the corresponding author upon reasonable request.

## Appendix A

**Table A1:** Survey Items and Response Options.

Survey Item	Response Options
This section of the questionnaire asks you to rate the usefulness of the content in the Chronic Pain Profile in the context of working with a patient who has chronic pain.	
The strategies used to manage pain are divided into 5 categories: medications, medical, physical, psychological, and self-initiated strategies. How informative are these categories in the context of pain management?	Not at all informative–Very informative (place a mark on the scale)
A level of use score is provided for each strategy listed based on the amount used and the recommended level of use. How helpful is the level of use score when thinking about modifying pain management strategies?	Not at all helpful–Very helpful (place a mark on the scale)
How useful is it to have a patient’s rating of how much a specific strategy helps them manage their pain?	Not useful at all–Very useful (place a mark on the scale)
How useful is it to have patient questions related to each strategy included in the pain management profile?	Not useful at all–Very useful (place a mark on the scale)
If a patient brought a pain management profile similar to the example to their appointment with you, how useful would you rate the profile?	Not useful at all–Very useful (place a mark on the scale)
Please comment on why you thought the profile would be useful or not useful.	(Open response)
How much would having a patient complete a profile before their appointment save time during the appointment?	Would not save any time–Would save substantial time (place a mark on the scale)
Please comment on why you thought the profile would or wouldn’t save time during an appointment.	(Open response)
This section of the questionnaire asks you to rate the information provided in the Chronic Pain Profile.	
How easy was it to understand the information on the Chronic Pain Profile?	Not easy at all–Very easy (place a mark on the scale)
Rank the sections of the Chronic Pain Profile from not at all easy to understand to the very easy to understand.	
Patient information	Not at all easy, somewhat easy, easy, quite easy, very easy
Types of strategy	Not at all easy, somewhat easy, easy, quite easy, very easy
Level of use	Not at all easy, somewhat easy, easy, quite easy, very easy
How much the strategy helps	Not at all easy, somewhat easy, easy, quite easy, very easy
Questions	Not at all easy, somewhat easy, easy, quite easy, very easy
Please provide any comments or suggestions that you have for the “Patient Information” section of the Profile.	(Open response)
Please provide any comments or suggestions that you have for the “Types of Strategies” section of the Profile.	(Open response)
Please provide any comments or suggestions that you have for the “Level of Use” section of the Profile.	(Open response)
Please provide any comments or suggestions that you have for the “How much the strategy helps” section of the Profile.	(Open response)
Please provide any comments or suggestions that you have for the “Questions” section of the Profile	(Open response)
This section of the questionnaire asks about your reactions to the Chronic Pain Profile web page. Click on the link to access the web page.	
How attractive would you rate the overall appearance of the web site?	Not at all attractive–Very attractive (place a mark on the scale)
How easy was the web page to use?	Not easy at all–Very easy (place a mark on the scale)
How easy was it to find the information that you wanted?	Not easy at all–Very easy (place a mark on the scale)
Please provide any feedback that you think might be helpful related to the appearance, function, or content of the web site.	(Open response)
This section of the questionnaire asks some general demographic questions about you.	
Your gender is:	Male, female
Your age on your last birthday was:	(Select age from list)
What is your race?	White, Black or African American, American Indian or Alaska Native, Asian, Native Hawaiian or other Pacific Islander, Hispanic or Latino
What is your marital status?	Married, Divorced or Widowed, Separated, Never Married, Living with Partner
In what state do you currently reside?	(Select State from list)
What is your current employment status?	Part-Time (less than 40 h per week), Full-Time (40 h or more per week), Retired, Unemployed
What is your primary site of professional practice?	Community Pharmacy (Chain), Community Pharmacy (Independent), Hospital Pharmacy (Inpatient), Hospital Pharmacy (Outpatient), Long-Term Care Pharmacy, Ambulatory Care Pharmacy, Other
For how many years have you actively practiced pharmacy?	(Select years from list)
How frequently do your job responsibilities involve helping patients manage their pain?	Almost never–About half the time–Almost all the time (place a mark on the scale)
Have you completed continuing education on pain management?	Yes, No
Do you currently or have you ever worked at a professional practice site that involves helping patients manage their pain (e.g., a pain clinic)?	I currently work at a site that helps patients manage pain; I previously worked at a site that helps patients manage pain; I do not currently, nor have I ever, worked at a site that helps patients manage pain
